# Right Temporoparietal Junction Involvement in Autonomic Responses to the Suffering of Others: A Preliminary Transcranial Magnetic Stimulation Study

**DOI:** 10.3389/fnhum.2020.00007

**Published:** 2020-01-28

**Authors:** Jonas G. Miller, Guohua Xia, Paul D. Hastings

**Affiliations:** ^1^Center for Mind and Brain, University of California, Davis, Davis, CA, United States; ^2^Department of Psychiatry and Behavioral Sciences, Stanford University, Stanford, CA, United States

**Keywords:** empathy, parasympathetic nervous system, psychophysiology, right temporoparietal junction, TMS

## Abstract

Functional neuroimaging studies have emphasized distinct networks for social cognition and affective aspects of empathy. However, studies have not considered whether substrates of social cognition, such as the right temporoparietal junction (TPJ), play a role in affective responses to complex empathy-related stimuli. Here, we used repetitive transcranial magnetic stimulation (TMS) to test whether the right TPJ contributes to psychophysiological responses to another person’s emotional suffering. We used a theory of mind functional localizer and image-guided TMS to target the sub-region of the right TPJ implicated in social cognition, and measured autonomic and subjective responses to an empathy induction video. We found evidence that TMS applied at 1 Hz over the right TPJ increased withdrawal of parasympathetic nervous system activity during the empathy induction (*n* = 32), but did not affect sympathetic nervous system activity (*n* = 27). Participants who received TMS over the right TPJ also reported feeling more irritation and annoyance, and were less likely to report feeling compassion over and above empathic sadness, than participants who received TMS over the vertex (*N* = 34). This study provides preliminary evidence for the role of right TPJ functioning in empathy-related psychophysiological and affective responding, potentially blurring the distinction between neural regions specific to social cognition vs. affective aspects of empathy.

## Introduction

Although empathy-related responses involve changes in multiple neurobiological systems, few studies have examined central and autonomic system integration in response to observing suffering in others (Hastings et al., 2014). Furthermore, social neuroscience has emphasized the distinctiveness of neural regions associated with cognitive vs. affective components of empathy-related responding, but psychological models have long considered these components to be interdependent (Zaki and Ochsner, 2012). For instance, perspective-taking could be one social-cognitive route to feeling empathy and compassion rather than aversive emotions (Batson et al., 1997; Vaish et al., 2009; Hastings et al., 2014). In this study we used transcranial magnetic stimulation (TMS) to test whether the functioning of one region centrally implicated in social cognition, the right temporoparietal junction (TPJ), is involved in instantiating autonomic and subjective aspects of responding to others’ emotional suffering.

Observing suffering in others can elicit changes in the activity of the parasympathetic and sympathetic branches of the autonomic nervous system (Hastings et al., 2014; Stellar et al., 2015; Miller, 2018). The parasympathetic nervous system downregulates arousal to contribute to a calm, soothed state that is conducive to social engagement (Porges, 2007). The sympathetic nervous system plays an important role in preparing the body for defensive responses to stress (Porges, 2007). In response to emotion induction video procedures, a dynamic pattern of parasympathetic activity characterized by initial decreases followed by rebound has been linked with subjective empathy, prosocial behavior, control of aggression, and positive maternal caregiving (Miller et al., 2013, 2016; Cui et al., 2015; Giuliano et al., 2015). Conversely, increased sympathetic reactivity to emotional stimuli contributes to increased stress-related arousal that undermines prosocial emotions and behaviors (Fabes et al., 1993; Kalvin et al., 2016). In addition, anger and annoyance, emotions that can interfere with compassionate and prosocial responses (Eisenberg, 2000), are typically associated with increased sympathetic and decreased parasympathetic activity (Kreibig, 2010). Taken together, flexible parasympathetic and decreased sympathetic nervous system reactions to others’ distress have been linked to prosocial responses. Conversely, increased sympathetic activity coupled with decreasing or inflexible parasympathetic activity may contribute to heightened arousal that interferes with prosocial emotions and behaviors.

In addition to physiological changes, observing suffering in others can elicit a range of subjective responses, including sharing or resonating with another person’s feelings (i.e., empathy) and feelings of concern coupled with a desire to alleviate the other’s suffering (i.e., compassion; Singer and Klimecki, 2014; Decety, 2015), but can also elicit feelings of annoyance that can lead to hostility. For example, harsh and abusive parents report being more annoyed and irritated by infant cries than non-abusive parents (Frodi and Lamb, 1980); mental health workers can experience increased irritability toward others’ needs as a symptom of work-related stress and burnout (Figley, 2002); and in research on public attitudes toward people with mental health problems, some people report emotional reactions of anger and irritation toward depressed individuals, who experience periods of intense and chronic suffering (Angermeyer and Matschinger, 2004).

Cognitive processes also can provide an understanding of someone else’s thoughts, intentions, and emotions. The cognitive process of taking another person’s perspective has been shown to be rooted in brain regions that are separate from those that support feeling empathy or compassion (Shamay-Tsoory et al., 2009; Kanske et al., 2015; Preckel et al., 2018), or feeling aggravated by others’ suffering (Kim et al., 2015). In particular, the right TPJ shows increased activation to functional magnetic resonance imaging (fMRI)-based social cognition tasks (Saxe and Powell, 2006; Völlm et al., 2006; Cheng et al., 2010), and stimulation of the right TPJ has been shown to interfere with performing tasks that require thinking about the mental states of others (Young et al., 2010; Mai et al., 2016; Coll et al., 2017). Thus, multiple neuroscience methods suggest that the right TPJ is a critical brain region for inferring and reasoning about others’ cognitive and emotional states.

Interestingly, the right TPJ has been consistently linked with distinguishing between self and other representations, or knowing that one’s own affective state is distinct from—albeit potentially due to—the affective state of another person (Decety and Lamm, 2007; Steinbeis, 2016; Lamm et al., 2019). One explanation for right TPJ involvement in perspective-taking and self-other distinction is that the two processes are interrelated. Self-other distinction is an important part of effectively taking another person’s perspective, as empathy and compassion require affective arousal without confusion over whose feelings belong to whom (Batson et al., 1987; de Vignemont and Singer, 2006; Decety and Lamm, 2009). Overlap in self-other representations may contribute to threat-related autonomic states (Buffone et al., 2017) as well as contagion of aversive effect (Decety and Lamm, 2009). Thus, perspective-taking with self-other distinction is a social cognitive process that could inherently regulate affective processes including psychophysiology. In addition, right TPJ activity during emotion introspection has been associated with trait-level affective empathy (Knight et al., 2019), further suggesting that right TPJ functioning may play a role in affective aspects of empathy. However, at the neural level, the potential role of the right TPJ in modulating physiological activity in response to others’ emotional suffering has yet to be examined.

Lastly, the overwhelming majority of neuroscience research on empathy-related responding has related brain activity during fMRI to behavioral measures. Scientists have argued for more brain stimulation studies on empathy using techniques like TMS to complement evidence from other neuroimaging methods (Hétu et al., 2012). Stimulating specific neural regions may provide experimental confirmation of their causal roles in distinct aspects of empathic engagement with others, such as cognitive vs. affective empathy. To date, TMS studies of empathy have almost exclusively focused on cognitive aspects of empathy (Yang et al., 2018). We are aware of only two published TMS studies of affect-related empathy processes; Balconi and Bortolotti (2012) and Balconi and Canavesio (2013) found that stimulation of prefrontal regions affected facial mimicry of, and subjective empathic responsiveness to, pictures of others’ emotional facial expressions. Whether TMS can provide evidence for the role of the right TPJ in autonomic responding to more complex social stimuli is unclear.

The current study used a multi-method approach to test whether right TPJ functioning is necessary for instantiating physiological aspects of empathic responding to others’ emotional suffering. To the extent that perspective-taking and self-other distinction are: (a) psychologically interdependent; (b) dependent on the right TPJ; and (c) important for effective physiological regulation, the functioning of the right TPJ was expected to play a role in promoting parasympathetic flexibility. Disruption of the right TPJ using TMS was expected to decrease parasympathetic flexibility or increase parasympathetic withdrawal, and potentially increase sympathetic nervous system activity, in response to others’ suffering. In a secondary, complementary analysis, we evaluated whether receiving TMS over the right TPJ was linked to experiencing less compassion and empathy and more annoyance/irritation during the emotion induction procedure.

## Materials and Methods

### Participants

This study initially included 41 right-handed young adults with normal or corrected-to-normal vision (30 females; Mean age = 21.47, *SD* = 3.33, range = 18–31). Seven participants were dropped from analyses due to refusing TMS or not participating in both sessions. Thus, the final sample included 34 participants (25 females; Mean age = 20.86, *SD* = 2.75, range = 18–30). Individuals were screened for contraindication to TMS (Rossi et al., 2009) and were excluded from participating if they met any of the following criteria: metal in the body that could not be removed, fear of small spaces, pregnant, history of neurological disorders or head trauma, history of seizures, significant visual or hearing impairment, developmental delays or psychiatric disorders, major medical problems, and taking prescription medications. Alcohol use was assessed (Saunders et al., 1993), and individuals who regularly engaged in heavy/binge drinking were also excluded. All participants provided written consent, and the study protocol was approved by the institutional review board of our university and carried out in compliance with the Declaration of Helsinki.

### Procedure

Each participant took part in two sessions that were on average 18 days apart (*SD* = 11.43). In the first session, participants underwent structural and functional MRI. This included a theory of mind task used to localize the right TPJ activity for each participant (Dodell-Feder et al., 2011). In the second session, electrodes were attached to the chest and back to measure parasympathetic and sympathetic nervous system activity using electrocardiograph (ECG) and impedance cardiograph signals. Participants were randomly assigned to receive 20 min of repetitive TMS either over the vertex (control condition; *n* = 17; 13 females; Mean age = 20.87, *SD* = 2.17, range = 18–26) or over the right TPJ (*n* = 17; 12 females; Mean age = 20.86, *SD* = 3.35, range = 18–30). After completing the TMS administration, participants first played a non-emotional computer game, then watched a neutral film clip, then a sadness induction film clip and reported on their subjective emotional experiences. Repetitive TMS leads to disruption of neural activity and behavioral effects that outlast the duration of TMS for roughly 50–200% of the duration of stimulation (Walsh and Cowey, 2000).

### Neutral Film

To assess whether TMS over the vertex vs. right TPJ had differential effects on physiology in general (i.e., not specific to the sadness induction), cardiac data were recorded while participants viewed a 2 min instructional film clip meant to be neutral in content. This film clip has been used in previous studies of emotion as a neutral baseline (Troy et al., 2013). The neutral film clip was presented on average 10.57 min after administration of TMS (*SD* = 3.66, range = 0.91–18.58). The neutral film clip was presented immediately before the emotion induction procedure.

### Emotion Induction Procedure

Cardiac data were recorded during a sadness induction procedure that involved watching a 2 min 39 s clip from *The Champ*, which has been used extensively to study physiological and subjective aspects of empathic sadness (Marsh et al., 2008; Hastings et al., 2009; Seider et al., 2011). This film clip presents a boy experiencing emotional distress in response to the death of his father after a boxing match. The video was presented on average 13.38 min after administration of TMS (*SD* = 3.54, range = 4.15–21.99). Following the video, participants were asked to rate on a 7-point Likert scale ranging from 1 (Not at all) to 7 (Extremely/a great deal) the degree to which they experienced different emotions during the film clip (afraid/scared, annoyed/irritated, anxious, compassion/sympathy, happy, sad, and warmth/tenderness). The emotion terms were presented in a random order across participants. Given that sadness was the primary emotion depicted in the film clip, we used ratings of sadness as a measure of subjective empathy. To measure compassionate and hostile emotional responding to others’ suffering, we used the ratings of compassion/sympathy and annoyed/irritated, respectively.

### MRI Procedure

At the first session, anatomical and fMRI data were acquired using a Siemens 3T Tim Trio scanner with a 32-channel head coil. A high-resolution anatomical image was obtained for each participant using an MPRAGE pulse sequence with a 2,500 ms repetition time (TR), 4.33 ms echo time (TE), 7° flip angle, 0.9 × 0.9 × 0.9 voxels, 208 slices, and 243 mm field-of-view (FOV). Functional images were acquired using a gradient echo pulse sequence with 2,000 ms TR, 27 ms TE, 80° flip angle, 3.5 × 3.5 × 3.5 mm voxels, 35 slices, and 224 mm FOV.

**Figure 1:**
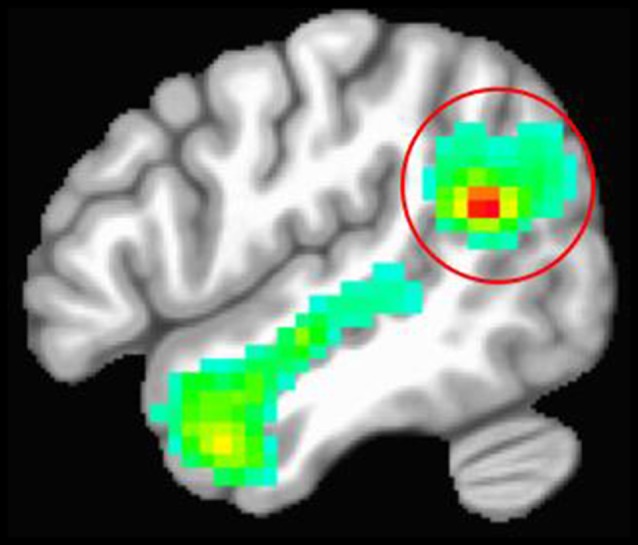
Average location of the right temporoparietal junction (TPJ) based on the theory of mind functional localizer task. The red circle is around the right TPJ cluster. The red portion of the cluster represents the peak activation for contrasting stories about beliefs vs. stories about photographs.

We used a functional localizer to identify each participant’s right TPJ. Participants underwent an fMRI task that presented false-belief stories (i.e., inferences about someone’s beliefs) vs. stories that required inferences about faulty information in photographs (Dodell-Feder et al., 2011). There were two runs of 10 trials including five false-belief stories and five false-photograph stories. Each trial consisted of a 14 s story and was separated by a 12 s fixation screen. This fMRI task was specifically designed to localize right TPJ activation related to social cognition (i.e., theory of mind; Dodell-Feder et al., 2011). Given that we were interested in testing whether the sub-region of the right TPJ implicated in social cognition is causally involved in empathy-related psychophysiology and affect, we chose to use this theory of mind task to guide TMS instead of an emotion induction paradigm. Figure 1 shows the average right TPJ location based on the functional localizer task.

Each participant’s data were preprocessed and analyzed using AFNI (Cox, 1996). Data were motion-corrected, registered to the first volume, and smoothed using a 6 mm half-maximum Gaussian kernel. BOLD response for each trial was modeled using a boxcar regressor of 14 s. The fixation cross was treated as the implicit baseline condition. For each participant, the right TPJ was defined based on a single subject whole-brain analysis of the contrast between false-belief stories and false-photograph stories. AFNI’s 3dClustSim (version AFNI_16.3.05, 2016) was used to identify the minimum cluster size for each participant’s right TPJ as a voxel-wise threshold of *p* = 0.001 to obtain an overall alpha of *p* < 0.01.

### TMS Procedure

In the second session, we used a Magstim Super Rapid TMS system with a figure-8 shaped coil air-cooled by a vacuum powered fan. In both the control and right TPJ group, low-frequency (1 Hz) TMS was applied for 20 min at 100% of each participant’s motor threshold. The motor threshold was determined by examining the minimum stimulator output required to elicit thumb movement for 50% of TMS pulses over the motor cortex (Varnava et al., 2011). We used Brainsight software (Rogue Industries, Standish, ME, USA) to localize TMS to the stimulation site based on each participant’s anatomical data (control group) or anatomical combined with functional MRI data (right TPJ group). For each individual in the control group, the vertex was defined as the meeting point between the left and right postcentral gyri (Ruff et al., 2006). The coil was placed tangentially against the scalp and oriented in an upright position with the handle pointing posteriorly. For the right TPJ group, each participant’s stimulation site was defined by their functional localizer task data coregistered with their anatomical data in native space. The coil was placed tangentially against the scalp and oriented with the handle pointing posteriorly and approximately 45 degrees to the central sulcus in the right TPJ group. Brainsight was used to monitor accurate coil position during TMS for both conditions.

### Psychophysiological Data

ECG and impedance data obtained during the neutral and emotion induction videos were processed using software from MindWare Technologies. Pre-Ejection period (PEP) was used as a measure of sympathetic nervous system activity. PEP is the time in milliseconds between cardiac ventricular depolarization and opening of the aortic valve. Shorter PEP reflects greater sympathetic nervous system activity. Due to not providing useable impedance data or technical problems, PEP data were missing for four participants in the right TPJ group and three participants in the control group. The root-mean-square of successive differences (RMSSD) was used as a measure of heart rate variability related to parasympathetic nervous system activity. Due to technical problems, RMSSD data were missing for 2 participants in the control group.

PEP and RMSSD values were computed for the duration of the neutral film clip and in four 40 s segments of the Champ film clip. The inspection of the data showed one participant with an extreme outlier value for RMSSD (greater than the mean by 1.5 times the difference between quartile 1 and 3). This participant’s RMSSD data was winsorized to maintain the rank-order within the sample by bringing their value down to the second-highest value and adding one.

### Analyses

We used mixed analysis of variance (ANOVA) models to examine the effects of applying TMS to the right TPJ on experiential and physiological responses to others’ suffering. This included planned comparisons of the effects of TMS on subjective feelings and overall patterns of change in RMSSD and PEP (i.e., linear vs. quadratic change) over the course of the film. All participants with available data were used for these analyses.

## Results

Descriptive statistics are presented in Table 1.

**Table 1:** Descriptive statistics of key study variables.

Variable	*n*	*M*	*SD*
RMSSD neutral video	32	49.59	27.53
PEP neutral video	31	102.00	16.39
RMSSD sad epoch 1	32	49.44	26.59
RMSSD sad epoch 2	32	45.97	19.51
RMSSD sad epoch 3	32	45.93	22.10
RMSSD sad epoch 4	32	44.34	26.29
PEP sad epoch 1	27	104.22	13.07
PEP sad epoch 2	27	106.22	12.83
PEP sad epoch 3	27	106.74	13.30
PEP sad epoch 4	27	107.33	13.43
Annoyance/Irritation	34	1.79	1.20
Compassion/Sympathy	34	5.76	1.26
Sadness	34	5.62	1.28

*Note. RMSSD, root mean square of successive differences; PEP, pre-ejection period*.

### Physiology During Neutral Film Clip

Participants in the right TPJ and vertex groups did not differ in terms of RMSSD or PEP during the neutral film clip (both |*t*| < 1.43, *p* > 0.163).

### Physiology During Sad Film Clip

To analyze the effect of TMS site on parasympathetic and sympathetic nervous system activity during the sadness induction video, a 2 (group: right TPJ vs. vertex) × 4 (epoch of champ video) mixed ANOVA was conducted for each of RMSSD and PEP. Mauchly’s test showed that the assumption of sphericity had been violated for RMSSD ($\chi_{\left(5\right)}^{2}$ = 29.02, *p* < 0.001) and PEP ($\chi_{\left(5\right)}^{2}$ = 32.00, *p* < 0.001). Thus, degrees of freedom were corrected using Greenhouse-Geisser estimates of sphericity (RMSSD *ε* = 0.66, PEP = *ε* = 0.56).

**Figure 2:**
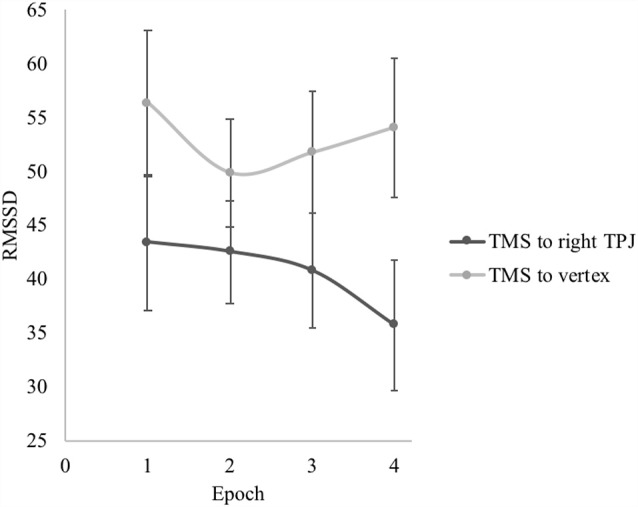
Effect of transcranial magnetic stimulation (TMS) site on root mean square of successive differences (RMSSD) during the sadness induction video. Error bars represent the standard errors of the means.

For RMSSD, there was no significant main effect of epoch, *F*_(1.97,59.03)_ = 1.78, *p* = 0.178, partial *η* = 0.06. There was a significant interaction effect of site of TMS on quadratic change in RMSSD over the course of the sadness induction video, *F*_(1,30)_ = 5.98, *p* = 0.021, partial *η* = 0.17 (see Figure 2). Participants who received TMS to the right TPJ showed a pattern of accelerating decreases in RMSSD as the intensity of the depicted sadness increased over the course of the video. This pattern was characterized by statistically nonsignificant decreases in RMSSD from epochs 1 to 2 (Mean difference = −0.87, *p* = 0.778) and 2 to 3 (Mean difference = −1.75, *p* = 0.397), and a significant decrease in RMSSD from epochs 3 to 4 (Mean difference = −5.01, *p* = 0.038). Looking at the overall change, participants who received TMS to the right TPJ decreased RMSSD from the beginning to the end of the sadness induction video (from epoch 1 to 4) at the trend level (Mean difference = −7.63, *p* = 0.084). Conversely, participants who received TMS to the vertex showed a pattern of initial decrease followed by accelerating rebound in RMSSD over the course of the sadness induction video, matching the patterns that have been previously observed in other samples (Cui et al., 2015; Miller et al., 2016). RMSSD at the beginning and end of the sadness induction video were not significantly different for participants who received TMS to the vertex (Mean difference = −2.24, *p* = 0.625). By epoch 4, RMSSD was significantly higher for participants in the vertex group than for those in the TPJ group, *t*_(30)_ = 2.07, *p* = 0.047.

**Figure 3:**
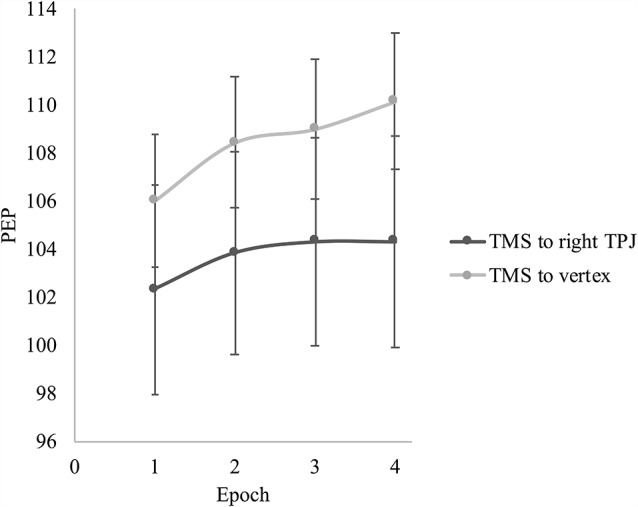
Effect of TMS site on pre-ejection period (PEP) during the sadness induction video. Error bars represent the standard errors of the means.

For PEP, there was a significant main effect of epoch, *F*_(1.69,42.29)_ = 9.25, *p* = 0.001, partial *η* = 0.27, but no interaction between epoch and TMS group, *F*_(1.69,42.29)_ = 1.00, *p* = 0.364, partial *η* = 0.04. For the whole sample, there were significant linear, *F*_(1,25)_ = 12.39, *p* = 0.002, partial *η* = 0.33, and quadratic changes, *F*_(1,25)_ = 4.57, *p* = 0.042, partial *η* = 0.16, in PEP suggesting a general trend of increasing PEP length (i.e., decreasing sympathetic nervous system activity) over the course of the video induction. Looking at the overall change from epoch 1 to 4, PEP was significantly longer by the end of the sadness induction video compared to the beginning (Mean difference = 3.07, *p* = 0.001), indicating that sympathetic nervous system activity had decreased over the course of the induction for participants in both TMS conditions. Figure 3 presents the change in PEP over the course of the sadness induction for participants who received TMS to the right TPJ vs. the vertex.

### Subjective Emotional Experience

Participants who reported feeling more annoyed/irritated in response to the sadness induction reported feeling less compassion/sympathy (whole sample *r* = −0.64, *p* < 0.001; right TPJ group *r* = −0.52, *p* = 0.033; vertex group *r* = −0.69, *p* = 0.002) and sadness (whole sample *r* = −0.44, *p* = 0.009; right TPJ group *r* = −0.60, *p* = 0.011; vertex group *r* = −0.53, *p* = 0.028). Ratings of compassion/sympathy and sadness were positively correlated (whole sample *r* = 0.50, *p* = 0.002; right TPJ group *r* = 0.50, *p* = 0.043; vertex group *r* = 0.88, *p* < 0.001).

**Figure 4:**
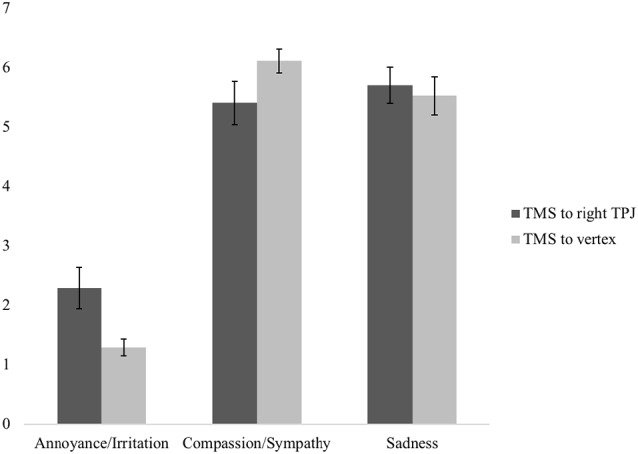
Effect of TMS site on reported feelings during the sadness induction video. Error bars represent the standard errors of the means.

Figure 4 presents the reports of subjective emotional experiences of participants who received TMS to the right TPJ vs. the vertex. To analyze the effect of TMS site on subjective emotion, we conducted a 2 (group: right TPJ vs. vertex) × 3 (emotion: annoyed/irritated vs. compassion/sympathy vs. sad) mixed ANOVA. Mauchly’s test showed that the assumption of sphericity had been violated ($\chi_{\left(2\right)}^{2}$ = 15.27, *p* < 0.001). Thus, degrees of freedom were corrected using Greenhouse-Geisser estimates of sphericity (*ε* = 0.72). A significant main effect of emotion, *F*_(1.44,46.08)_ = 105.00, *p* < 0.001, partial *η* = 0.77, was subsumed within a significant interaction between TMS group and emotion, *F*_(1.44,46.08)_ = 3.77, *p* = 0.044, partial *η* = 0.11 (see Figure 1). In response to the sadness induction video, participants who received TMS to the right TPJ reported significantly more annoyance/irritation compared to participants who received TMS to the vertex (*p* = 0.013). The two groups did not differ in their reports of compassion (*p* = 0.102) or sadness (*p* = 0.690).

Although the two groups did not differ in their mean levels of compassion and sadness, there were significant differences between participants in the two TMS groups in the relative strengths of their affective arousal, specifically in their reported experiencing of annoyance/irritation relative to compassion/sympathy, *F*_(1,32)_ = 5.90, *p* = 0.021, partial *η* = 0.16, and compassion/sympathy relative to sadness, *F*_(1,32)_ = 5.34, *p* = 0.027, partial *η* = 0.14. Participants who received TMS to the vertex reported more compassion/sympathy relative to annoyance/irritation than participants who received TMS to the right TPJ (*p* = 0.021). In addition, participants who received TMS to the vertex reported significantly more compassion/sympathy than sadness (*p* = 0.037), whereas participants who received TMS to the right TPJ reported similar levels of compassion/sympathy and sadness (*p* = 0.284).

Taken together, participants who received TMS to the right TPJ had a profile of feeling more irritation/annoyance than participants in the control group. The right TPJ group reported similar levels of compassion/sympathy and sadness, whereas the control group reported feeling more compassion/sympathy than sadness. Participants in the control group also reported a larger difference in their ratings of compassion/sympathy relative to annoyance/irritation than the participants in the right TPJ group.

## Discussion

This preliminary study suggests that disrupting right TPJ activity with offline TMS affects physiological, and potentially some aspects of experiential, responses to others’ suffering. In response to a film clip depicting a child experiencing loss and sadness, TMS over the right TPJ appeared to lead to the withdrawal of parasympathetic nervous system activity. Conversely, participants who received TMS to the vertex demonstrated a pattern of parasympathetic flexibility (i.e., an initial decrease followed by rebound in parasympathetic activity) consistent with what has been observed in other samples (Cui et al., 2015; Miller et al., 2016). The right TPJ is widely considered an important neural region for social cognition, but our findings provide evidence that the right TPJ may also play a role in autonomic and affective responses to others’ suffering.

Right TPJ functioning appears to be important in modulating autonomic reactivity to others’ emotional suffering. These findings were not due to differences in overall arousal, as the two TMS groups did not differ in their physiological responses to a neutral film clip. In response to the sad film clip, TMS to the right TPJ induced a pattern of decreasing parasympathetic activity. The control group demonstrated a specific dynamic pattern of initially decreasing followed by rebounding parasympathetic activity. Previous studies have associated this pattern of parasympathetic flexibility with greater prosociality (Cui et al., 2015; Miller et al., 2016). One interpretation of this pattern of parasympathetic activity is that it reflects a sequence of first orienting to the other’s distress *via* some arousal (decreasing HRV) followed by calm, social engagement with that distress (HRV rebound; Hastings and Miller, 2014; Miller, 2018). The parasympathetic nervous system is considered to be a key part of a social engagement system, whereas autonomic arousal driven by the sympathetic nervous system can contribute to defensive responding (Porges, 2007). Interestingly, we did not find evidence that TMS to the right TPJ affected the sympathetic nervous system responding. Taken together, TMS to the right TPJ appeared to produce parasympathetic responses previously linked to less concern for others and less prosocial behavior. One interpretation of these findings is that the social cognitive processes supported by the right TPJ have downstream consequences for organizing parasympathetic responses in empathy contexts. These findings inform brain-body models of responding to others’ suffering by highlighting potential pathways by which central and autonomic systems interact. Right TPJ and parasympathetic functioning may be integrated central and autonomic components, respectively, of a social engagement system.

In addition to autonomic responding, the right TPJ may play a role in avoiding feeling aggravated by others’ suffering. One interpretation of this finding is that social cognitive processes supported by the right TPJ contribute to effective emotion regulation, including inhibition of feelings that can lead to hostility or avoidance of others’ distress. TMS to the right TPJ did not affect levels of empathic sadness, which is in line with the perspective that dissociable networks are implicated in cognitive and affective aspects of empathy (Preckel et al., 2018). However, the right TPJ group reported feeling similar levels of compassion and empathic sadness, whereas participants in the control group reported feeling more compassion than empathic sadness. It has been argued that compassion reflects feeling *for* another in need or distress and that empathy reflects feeling *with* or similar to another (Singer and Klimecki, 2014). The right TPJ has consistently been linked to perspective-taking with self-other distinction, and these social cognitive processes may contribute to experiencing compassion above and beyond empathic sadness. Although compassion and empathy are generally both considered emotional mechanisms of prosociality (de Waal, 2008; Davidov et al., 2016), compassion is more strongly linked to positive social and personal outcomes like prosocial behavior toward others (Jordan et al., 2016) and increased positive affect (Klimecki et al., 2013). Thus, participants who received TMS to the vertex reported experiencing an emotional profile that may be more positive and prosocial than the profile of emotions experienced by people who received TMS to the right TPJ. Whether these observed emotional profiles: (a) are due to TMS undermining social cognition; and (b) actually lead to different behavioral outcomes, are still open questions. A number of studies have posited that the link between the right TPJ and prosocial behavior is due to perspective-taking skills (Telzer et al., 2011; Morishima et al., 2012). Future research should test the possibility that downstream effects of right TPJ functioning on affective processing might mediate the link between social cognition and prosocial behavior.

There are a number of limitations of this preliminary study that should be considered. First, behavioral measures of perspective-taking or self-other distinction were not included. Given that these social cognitive processes are rooted in right TPJ functioning, presumably they were altered by disrupting the right TPJ activity. However, future research that includes these measures are necessary to confirm whether these social cognitive processes mediate the link between right TPJ functioning and affective response to others’ suffering. Second, a larger sample may have been needed to detect effects on compassion and sympathetic nervous system functioning than were evident for annoyance/irritation and parasympathetic functioning. In addition, applying TMS to the right TPJ and a control region within the same participants across multiple sessions and examining changes in affective responding would be a strong replication and extension of the present preliminary study. Third, we did not collect baseline ratings of subjective feelings. Thus, although we asked participants to report on their feelings during the empathy-induction video, we could not directly test whether group differences in subjective feelings were specific to this period. TMS over the TPJ generally produces more discomfort during stimulation than TMS over the vertex. This could have contributed to the TPJ group reporting greater annoyance, but it should be noted that the empathy-induction video and emotion ratings were administered, on average, more than 10 min after the conclusion of TMS. Fourth, the right TPJ may consist of an anterior portion that is more involved in reorienting attention and a posterior portion dedicated to social cognition (Krall et al., 2015). We cannot rule out that TMS in our study did not partially stimulate the more anterior region of the right TPJ and that this could have contributed to some of the findings. However, we used a theory of mind functional localizer and image-guided TMS to target the specific region of the right TPJ implicated in social cognition for each participant. Lastly, although the current study focused on the right TPJ functioning, spreading of stimulation to other regions strongly connected to the TPJ likely also occurred (Valero-Cabré et al., 2005). The right TPJ is connected to other regions implicated in social cognitive processes, including the precuneus, posterior cingulate, middle temporal gyrus, temporal pole, and medial prefrontal cortex (Krall et al., 2015). Although the effects of TMS on neural activity are strongest in the targeted region, indirect activation in other regions connected to the right TPJ may have also contributed to our findings.

There is a large literature that suggests that the right TPJ is important for social cognition, but the present findings link right TPJ functioning to physiological and subjective components of emotion in an empathy-induction task. We interpret the observed effects of TMS in this study as preliminary evidence that the right TPJ contributes to physiological and experiential aspects of responding to others’ suffering. The right TPJ is widely considered a core part of a social cognition network that is distinct from networks that support empathic sharing of emotion and compassion. At the same time, it is important to keep in mind that social cognitive and affective processes are strongly interactive. Right TPJ functioning may be important for affective processes in response to complex social stimuli, but more brain stimulation research is necessary to replicate and build on these preliminary findings.

## Data Availability Statement

The dataset that was analyzed for this study can be found at https://osf.io/t5dw7/.

## Ethics Statement

The studies involving human participants were reviewed and approved by UC Davis Institutional Review Board. The patients/participants provided their written informed consent to participate in this study.

## Author Contributions

JM, GX, and PH designed the study. JM collected, processed, and analyzed the data. JM drafted the manuscript. GX and PH provided critical revisions. All authors approved the final version of the manuscript.

## Conflict of Interest

The authors declare that the research was conducted in the absence of any commercial or financial relationships that could be construed as a potential conflict of interest.
